# Diagnostic value of magnetic resonance imaging for patients with periprosthetic joint infection: a systematic review

**DOI:** 10.1186/s12891-023-06926-5

**Published:** 2023-10-09

**Authors:** Chang Shufen, Liu Jinmin, Zhang Xiaohui, Geng Bin

**Affiliations:** 1 Department of Orthopaedics, Lanzhou University Second Hospital, 730000 Lanzhou, Gansu China; 2 Gansu Province Clinical Research Center for Orthopaedics, Lanzhou, Gansu China

**Keywords:** Magnetic resonance imaging, Periprosthetic Joint infection, Diagnosis, Total joint arthroplasty

## Abstract

**Purpose:**

The purpose of this study was to provide a critical systematic review of the role of magnetic resonance imaging (MRI) as a noninvasive method to assess periprosthetic joint infections (PJIs).

**Methods:**

The electronic databases PubMed and EMBASE were searched, since their inception up to March 27, 2022. The included studies evaluated the reproducibility and accuracy of MRI features to diagnose PJIs. The article quality assessment was conducted by the COnsensus-based Standards for the selection of health Measurement INstruments (COSMIN) and Quality Assessment of Diagnostic Accuracy Studies-2 (QUADAS-2).

**Results:**

Among 1909 studies identified in the initial search, 8 studies were eligible for final systematic review. The included studies evaluated the reproducibility and accuracy of MRI features to diagnose PJIs. Seven of 8 studies showed good to excellent reliability, but only one article among them in which accuracy was evaluated had a low risk of bias. The intraclass correlation coefficient (ICC) and Cohen coefficient (κ) varied between 0.44 and 1.00. The accuracy varied between 63.9% and 94.4%. Potential MRI features, such as lamellated hyperintense synovitis, edema, fluid collection, or lymphadenopathy, might be valuable for diagnosing PJIs.

**Conclusion:**

The quality of the evidence regarding the role of MRI for PJIs diagnosis was low. There is preliminary evidence that MRI has a noteworthy value of distinguishing suspected periprosthetic joint infection in patients with total knee arthroplasty or total hip arthroplasty, but the definition of specific MRI features related to PJIs diagnosis lacks consensus and standardization. Large-scale studies with robust quality were required to help make better clinical decisions in the future.

**Supplementary Information:**

The online version contains supplementary material available at 10.1186/s12891-023-06926-5.

## Introduction

Total joint arthroplasty (TJA) has become the most common standard treatment for severe end-stage hip or knee disease, allowing joint pain relief, improvement of physical activity, and an increase in quality of life [1–4]. Although the postsurgical outcomes are usually excellent [5–8], the incidence of various complications will continue to increase over time, in large part due to the rise in the number of TJA over recent years and the increased life expectancy [9, 10]. Among these, periprosthetic joint infections (PJIs) is devastating because of prolonged hospitalization, repeated surgical interventions, or severe psychological and economic burden to patients [11, 12].

Determining the presence of PJIs remains a challenge of modern orthopedics as there is no gold standard diagnostic tool [13, 14]. In the last decade, the commonly used diagnostic criteria for PJIs were released by the European Bone and Joint Infection Society (EBJIS), Musculo-Skeletal Infection Society (MSIS) or two International Consensus Meetings on PJIs in 2013 (ICM 2013) and 2018 (ICM 2018) [15–17]. In general, the diagnostic approach in patients with suspected PJIs involves clinical findings, laboratory evaluation, radiology, biopsies with microbiological analysis, nuclear imaging, or intraoperative findings [18, 19]. There is no clear consensus about the choice of the most accurate imaging technique to detect suspected PJIs [20], especially in the case of a challenging diagnosis of an early or low-virulence infection.

Since the development of advanced metal artifact reducing techniques, magnetic resonance imaging (MRI) has been increasingly recognized as a noninvasive and valuable method in the evaluation of patients with septic arthritis [21] or hip and knee pain after arthroplasty [22–25]. However, there are two issues with the MRI diagnostic value of PJIs: (1) To date, there is no consensus on the diagnostic value of MRI for PJIs in total hip arthroplasty (THA) or total knee arthroplasty (TKA) patients [26–33]; and (2) There are no consistent criteria for the identification or definition of specific MRI features related to PJIs diagnosis. Consequently, it is necessary to systematically evaluate the diagnostic value of MRI features for PJIs.

This systematic review aimed to analyze the main value of MRI for PJIs diagnosis and summarize various helpful MRI appearances in identifying infected prostheses for THA or TKA patients.

## Materials and methods

This systematic review strictly adheres to the Preferred Reporting Items for Systematic Review and Meta-Analyses (PRISMA) guidelines [34]. Ethics committee approval was not needed to conduct a systematic review of the published literature.

### Search strategy

In March 27, 2022, a systematic literature search of the PubMed (Medline) and EMBASE (Elsevier) databases was conducted to identify the original studies that reported the imaging features of MRI for the diagnosis of PJIs. The detailed search terms were as follows: (Periprosthetic Infection OR Infected OR painful OR symptomatic) AND (THA OR TKA OR TJA OR TKR OR THR OR Knee Arthroplasty OR Hip Arthroplasty) AND (MRI OR MR OR MR Imaging OR magnetic resonance imaging) AND (Hip OR knee). The bibliographies of the included studies were also hand-screened to expand the search extent and to avoid missing relevant articles. Moreover, there were no search date limits in this study.

### Inclusion and exclusion criteria

After inspection for duplicates, studies were included based on the following inclusion criteria: (1) original articles in a peer-reviewed journal; (2) human studies; (3) reports on the features of MRI for the diagnosis of PJIs after THA or TKA; and (4) original articles in English. Studies were excluded if any of following criteria were satisfied: (1) review articles; (2) meta-analyses; (3) letters to the editor; (4) replies; (5) comments; (6) conference abstracts; (7) editorials; (8) case reports; (9) non-English studies; and (10) studies involving only animals.

### Study selection and data extraction

The eligible articles were independently selected by two reviewers according to title and abstract assessment. The final decision regarding inclusion was based on the full-text articles. If consensus was not reached in case of disagreement, a third reviewer was included.

The following study characteristics were extracted from the eligible studies: (1) authors; (2) year of publication; (3) study design; (4) number of subjects; (5) sex; (6) age; (7) prosthesis; (8) number of prostheses (total/infected/noninfected); (9) MRI setting; (10) duration from THA or TKA to MRI examination; and 11) study outcomes, including interrater and intrarater reliability (intraclass correlation coefficient (ICC) for continuous variables and Cohen coefficient (κ) for categorical variables with standard errors), sensitivity, specificity, positive predictive value (PPV), negative predictive value (NPV) and accuracy. Data extraction was conducted independently by the two reviewers, and any disputes between them were resolved by a consensus meeting.

### Methodologic quality appraisal and analysis

The included articles evaluated the reproducibility and accuracy of MRI features to diagnose PJIs. To assess the quality of these articles, the COnsensus-based Standards for the selection of health Measurement Instruments (COSMIN) tool and Quality Assessment of Diagnostic Accuracy Studies-2 (QUADAS-2) tool were used.

The reproducibility of the included articles in this study can be evaluated by reliability assessment using COSMIN reliability box 6 [35]. Reliability box 6 contains 3 domains: design requirements, statistical methods and other flaws [35]. Each standard is answered by the four point rating system (inadequate, doubtful, adequate, or very good) [36], and the final rating is determined by the lowest score given for any of the standards in box 6 (the worst score counts method) [37]. Interrater or intrarater reproducibility can be calculated with κ or ICC. The κ statistic was interpreted as follows: almost perfect agreement (0.81-1), substantial agreement (0.61–0.80), moderate agreement (0.41–0.60), fair agreement (0.21–0.40), slight agreement (0.01–0.20), and no agreement (0) [38]. The definition of ICC values was as follows: excellent reliability (> 0.90), good reliability (0.75–0.90), moderate reliability (0.50–0.75), and poor reliability (< 0.50) [39].

The QUADAS-2 tool is recommended for use in rating bias and applicability of a majority of diagnostic accuracy studies [40]. The QUADAS-2 contains 4 domains: patient selection, index test, reference standard, and flow and timing. Each question can be assessed with “low risk of bias”, “high risk of bias”, or “unclear risk” [41]. Moreover, sensitivity, specificity, PPV, NPV and accuracy were also calculated for each MRI feature.

## Results

### Search results

**Fig. 1:**
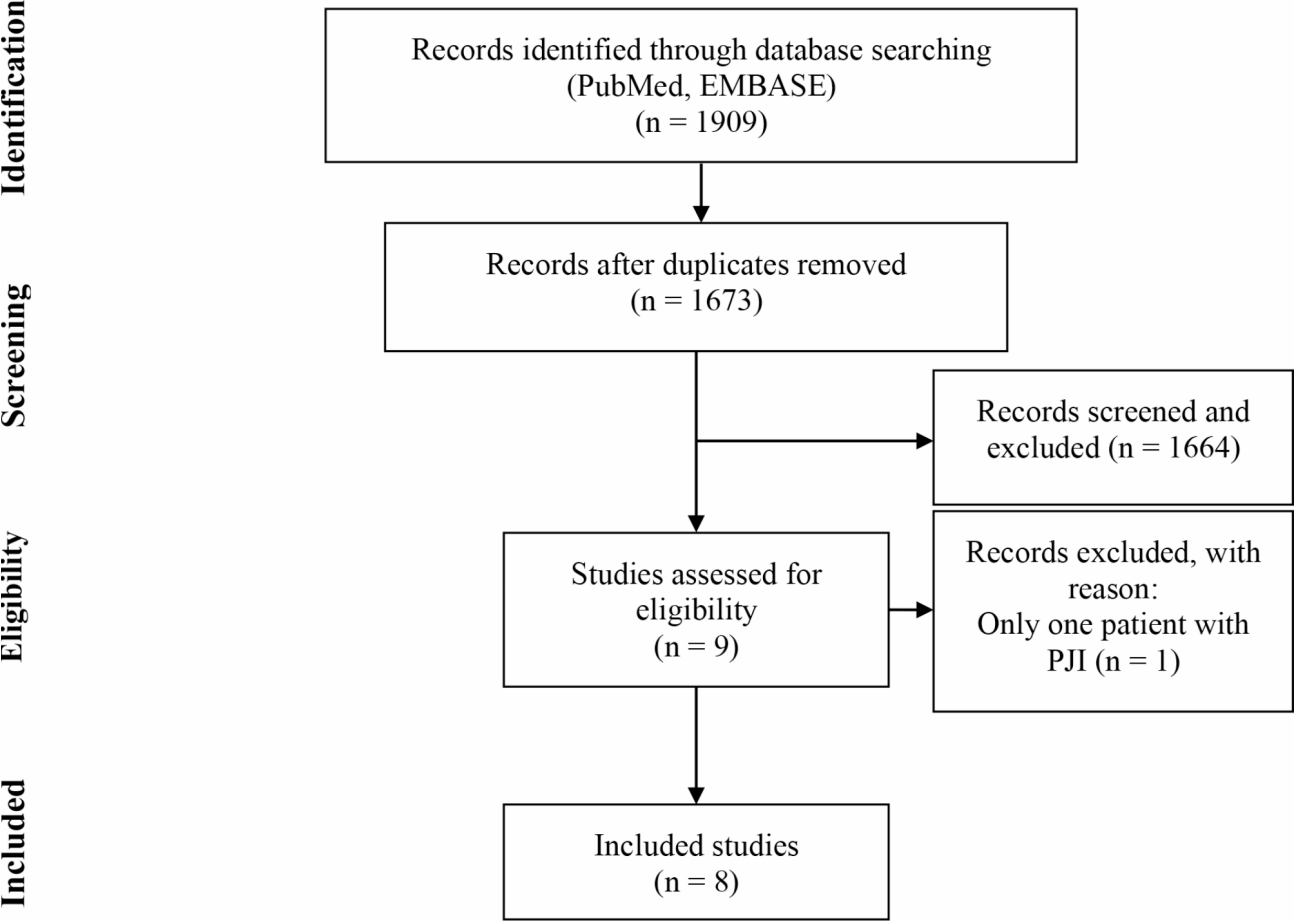
Flowchart of the literature systematic search

A flowchart of study selection is shown in Fig. 1. The systematic search strategy identified 1909 articles from PubMed and EMBASE. After removing 236 duplicate articles, 1673 articles remained. Of these, 1664 were excluded after analyzing the information in the title and abstract, while the remaining 9 full-text articles were downloaded for a further assessment. One article was excluded because it included only one patient with PJIs [42]. No other potentially relevant studies were extracted from the bibliographies of these articles. Finally, 8 eligible articles, which included a total of 645 patients, were summarized and analyzed in this study [26–33].

### Characteristics of included studies

The detailed study characteristics are summarized in Table 1. The included studies were published in 2013 (1/8) [33], 2014 (1/8) [31], 2016 (2/8) [30, 32], 2020 (3/8) [27–29] and 2021 (1/8) [26]. The study design was retrospective in 87.5% (7/8) of studies [26–30, 32, 33] and prospective in 12.5% (1/8) [31]. The number of subjects ranged from 30 to 140 patients. Six studies detected the diagnostic role of MRI for THA patients [26–31] and the remaining two studies focused on the MRI features of TKA patients [32, 33]. Altogether, a total of 645 patients, 206 (31.94%) with and 439 (68.06%) without PJIs, were assessed in 481 (74.57%) hip prostheses and 164 (25.43%) knee prostheses. MRI was performed by 1.5 T scanners in all eight studies.

**Table 1:** Study Characteristics

Study	Year	Design	Subjects	Male/female ratio	Age(mean ± SD or mean+;range)	Prosthesis	Number of prostheses (total/infected/noninfected)	MRI (Tesla; sequence)	Follow-up
1.Albano et al. [26]	2021	Retrospective, case control	119	53/66	66.9 ± 12.4	THA	119/38/81	1.5T; MARS + STIR	NA
2. Galley et al. [27]	2020	Retrospective, case control	140	68/72	69 ± 11^a^; 67 ± 11^b^	THA	140/40/100	1.5T; SEMAC + STIR	> 6 weeks
3. Gao et al. [28]	2020	Retrospective, case control	50	26/24	60 ± 13.6^a^;65 ± 13.2^b^	THA	50/25/25	1.5T; SEMAC	51.7–85.0 months (mean)
4. Schwaiger et al. [29]	2020	Retrospective, case control	30	12/18	66.4 ± 9.6	THA	30/15/15	1.5T; STIR + VAT	NA
5. Jiang et al. [30]	2016	Retrospective, case control	86	34/52	67 (range, 30–89)	THA	86/19/67	1.5T; FSE + VAT	NA
6. He et al. [31]	2014	Prospective	56	19/37	67 (range, 38–83)	THA	56/18/38	1.5T; FSE + STIR	NA
7. Li et al. [32]	2016	Retrospective, cross-sectional	108	43/65	64 (range, 43–87)	TKA	108/23/85	1.5T; FSE + IR + MAVRIC	> 1year
8. Plodkowski et al. [33]	2013	Retrospective, case control	56	29/27	65.8 ± 10.4^a^;65.7 ± 11.6^b^	TKA	56/28/28	1.5T; FSE + STIR	NA

THA: total hip arthroplasty; TKA: total knee arthroplasty; MARS: metal artifact reduction sequence; STIR: short tau inversion recovery; SEMAC: slice encoding for metal artifact correction; VAT: view angle tilting; FSE: fast-spin-echo; IR: inversion recovery; MAVRIC: multiacquisition variable resonance image combination; NA: not available; ^a^: PJI group; ^b^: control group

### Study quality appraisal and analysis

For reproducibility assessment (Table 2), seven studies were scored adequate to very good by the COSMIN reliability box [26–30, 32, 33], and only one study was scored inadequate [31]. Nevertheless, approximately 12.5% of the included articles did not analyze interrater reliability, and 50% of the included studies did not provide intrarater reliability assessment.

**Table 2:** Reproducibility of MRI Measurements to Diagnose Periprosthetic Joint Infection

Study (year)	MRI measurement	Interrater reliability K (95% CI)	Intrarater reliability K (95% CI)	COSMIN (reliability)
Total hip arthroplasty				
1. Albano et al. (2021) [26]	Effusion	K = 0.948 (NA)-0.966 (NA)	NA	+
	Synovitis	K = 0.858 (NA)-0.884 (NA)	NA	
	Lamellated synovitis	K = 0.855 (NA)-0.907(NA)	NA	
	Extracapsular edema	K = 0.905 (NA)-0.923 (NA)	NA	
	Bone edema	K = 0.927 (NA)	NA	
	Extracapsular collections	K = 0.920 (NA)-0.941 (NA)	NA	
	RNS	ICC = 0.98 (NA)	NA	
	DNS	ICC = 0.98 (NA)	NA	
	RNN	K = 0.99 (NA)	NA	
	DNN	K = 0.99 (NA)	NA	
2. Galley et al. (2020) [27]	Periosteal reaction, shaft	K = 0.79 (0.65–0.90)-0.95 (0.87-1.00)	NA	++
	Capsule edema	K = 0.88 (0.77–0.95)	NA	
	Intramuscular edema			
	Overall	K = 0.88 (0.78–0.95)	NA	
	Along surgical approach	K = 0.73 (0.59–0.85)	NA	
	Nonsurgical approach	K = 0.87 (0.76–0.95)	NA	
	Fluid collection			
	Intramuscular (subfascial), surgical approach	K = 0.77 (0.62–0.88)	NA	
	Intramuscular (subfascial), nonsurgical approach	K = 0.68 (0.42–0.81)	NA	
	Articular communication	K = 0.76 (0.61–0.88)	NA	
	Septation	K = 0.74 (0.59–0.88)	NA	
3. Gao et al. (2020) [28]	lamellated hyperintense synovitis	K = 0.76 (0.58–0.94)	K = 0.44 (0.19–0.69)-0.48 (0.23–0.72)	++
4. Schwaiger et al. (2020) [29]	Assessed for the whole region			++
	STIR signal hyperintensity in adjacent soft tissue indicating edema	K = 0.955 (0.749-1.000)	K = 1.000 (0.642-1.000)	
	Abscess anywhere in adjacent soft tissue	K = 1.000 (0.759-1.000)	K = 1.000 (0.582-1.000)	
	Enlarged inguinal or pelvic lymph nodes	K = 0.844 (0.638-1.000)	K = 1.000 (0.642-1.000)	
	Long axis of largest lymph node ≥ 17 mm	NA	NA	
5. Jiang et al. (2016) [30]	Soft tissue mass	K = 0.814 (NA)	NA	+
	Soft tissue edema	K = 0.791 (NA)	NA	
	Bone destruction	K = 0.826 (NA)	NA	
	Fistula	K = 0.884 (NA)	NA	
6. He et al. (2014) [31]	Thickened hyperintense synovium, extracapsular soft-tissue and bone edema, local lymphadenopathy, and extracapsular collections	NA	NA	
Total knee arthroplasty				
7. Li et al. (2016) [32]	lamellated hyperintense synovitis	K = 0.82 (0.72–0.91)	K = 0.83 (0.74–0.93)	++
8. Plodkowskiet al (2013) [33]	lamellated hyperintense synovitis	K = 0.82 (0.72–0.93)	K = 0.89 (0.78-1.00)	++

COSMIN: COnsensus-based Standards for the selection of health Measurement Instruments; K: Cohen’s Kappa; ICC: intraclass correlation coefficient; RNS: ratio of nodal size; RNN: ratio of node number; DNS; difference of nodal size; DNN: difference of node number. ++: very good;+: adequate; +/-: doubtful; -: inadequate; NA: not applicable

**Fig. 2:**
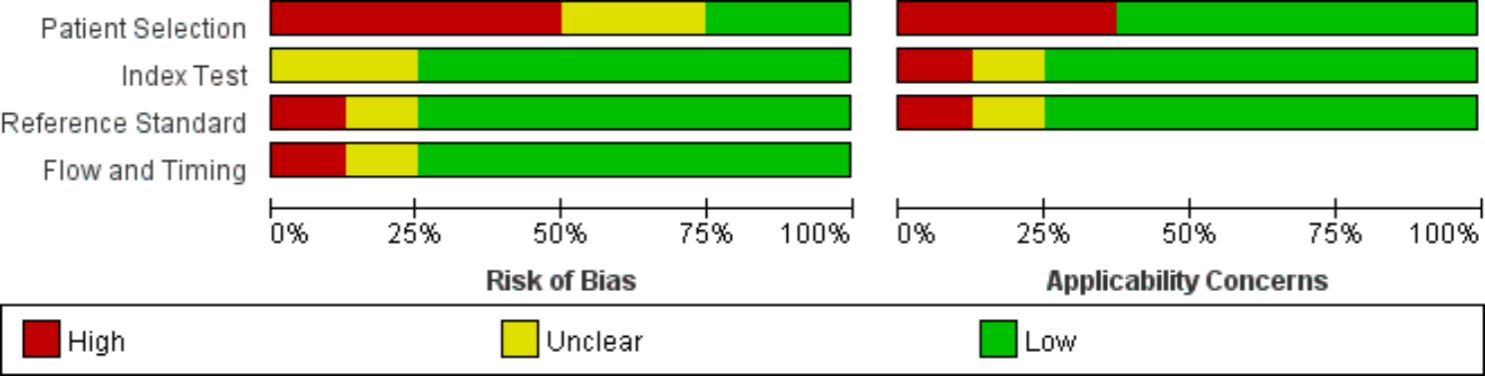
The methodologic quality of the included studies using QUADAS-2 shows the proportions of studies with high, low, or unclear risk of bias and concerns regarding applicability

**Fig. 3:**
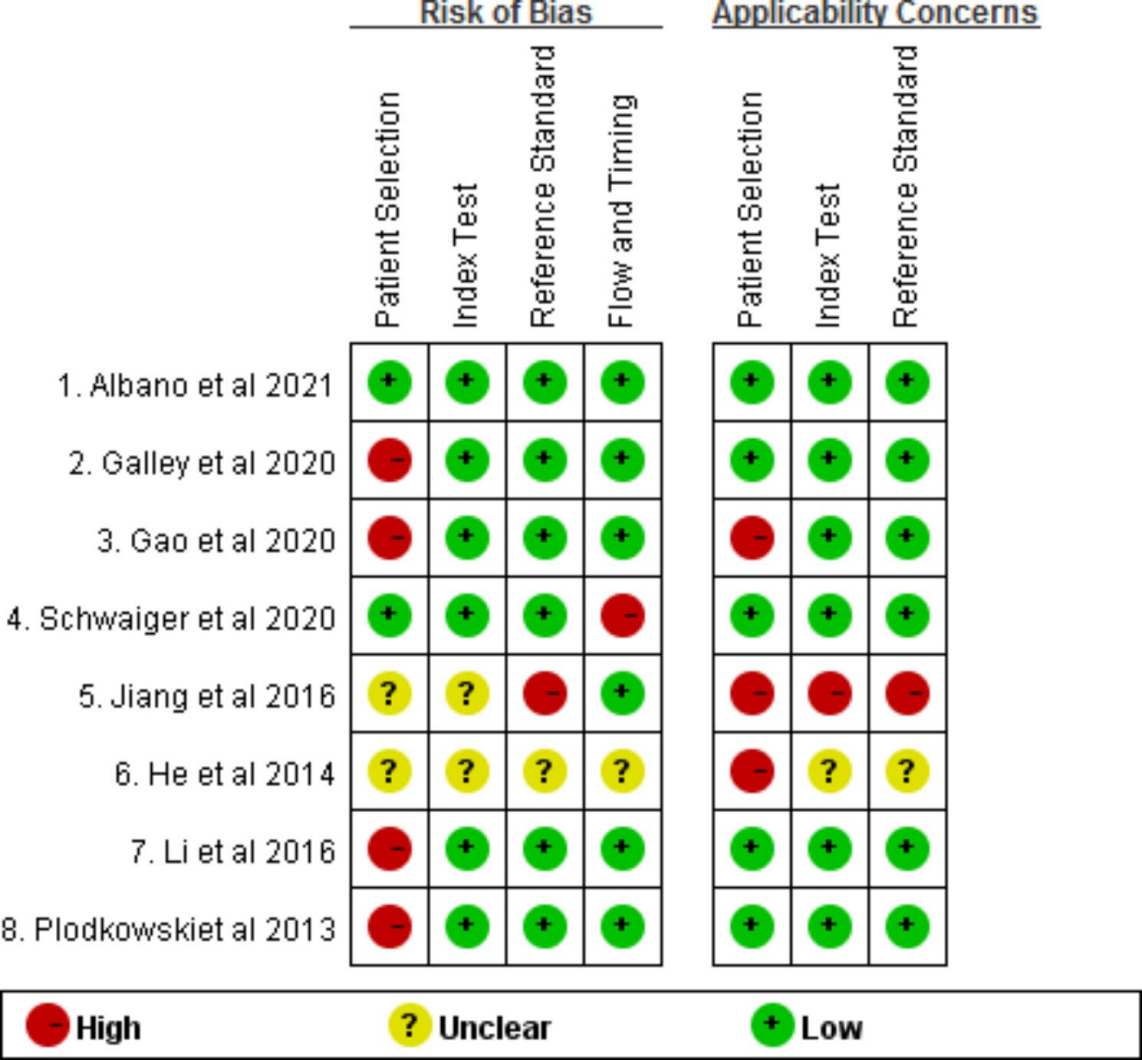
The methodologic quality of the included studies using QUADAS-2 shows each domain of studies with high, low, or unclear risk of bias and concerns regarding applicability

For accuracy assessment (Figs. 2 and 3), the methodological quality of seven studies had a high risk of bias [27–33], and only one study had a low risk of bias [26]. Because only one retrospective study scored a low risk of bias [26], the accuracy of the included articles showed more concerns regarding patient selection. Generally, the retrospective study design property will increase susceptibility to selection bias. In addition, the majority of included studies provide necessary information in regard to index test, reference standard, or flow and timing [26–29, 32, 33].

### MRI findings and PJIs

As shown in Tables 2 and 3, MRI features demonstrated high diagnostic performance in evaluating suspected PJIs, but the individual MRI signs of PJIs around the prosthesis varied or were inconsistent among all included studies. The important MRI findings of PJIs are summarized as follows:

**Table 3:** Accuracy of MRI Measurements to Diagnose Periprosthetic Joint Infection

Study (year)	MRI measurement	sensitivity	specificity	PPV	NPV	Accuracy
Total hip arthroplasty						
1. Albano et al. (2021) [26]	Effusion	76.3%	58.0%	46.0%	83.9%	63.9%
	Synovitis	47.4%	97.5%	90.0%	79.8%	81.5%
	Lamellated synovitis	26.3%	97.5%	83.3%	73.8%	74.8%
	Extracapsular edema	68.4%	86.4%	70.3%	85.4%	80.7%
	Bone edema	76.3%	81.5%	65.9%	88.0%	79.8%
	Extracapsular collections	50.0%	77.8%	51.4%	76.8%	68.9%
	RNS (1.106)	78.9%	76.5%	NA	NA	84.8%
	DNS (2.5 mm)	78.9%	77.8%	NA	NA	86.7%
	RNN (1.031)	89.5%	87.7%	NA	NA	93.1%
	DNN (0.5)	89.5%	87.7%	NA	NA	92.9%
2. Galley et al. (2020) [27]*	Periosteal reaction, shaft	78%	90%	76%	91%	86%
	Capsule edema	83%	95%	87%	93%	91%
	Intramuscular edema					
	Overall	95%	86%	73%	98%	89%
	Along surgical approach	78%	91%	78%	91%	87%
	Nonsurgical approach	90%	94%	86%	96%	93%
	Fluid collection					
	Intramuscular (subfascial), surgical approach	58%	93%	77%	85%	83%
	Intramuscular (subfascial), nonsurgical approach	28%	98%	85%	77%	78%
	Articular communication	58%	92%	74%	84%	82%
	Septation	58%	96%	85%	85%	85%
3. Gao et al. (2020) [28]*	lamellated hyperintense synovitis	80%	84%	83%	81%	82%
4. Schwaiger et al. (2020) [29]	Assessed for the whole region					
	STIR signal hyperintensity in adjacent softtissue indicating edema	87%	80%	81%	86%	83%
	Abscess anywhere in adjacent soft tissue	58%	100%	100%	64%	76%
	Enlarged inguinal or pelvic lymph nodes	93%	47%	64%	47%	70%
	Long axis of largest lymph node ≥ 17 mm	80%	87%	86%	81%	83%
5. Jiang et al. (2016) [30]	Soft tissue mass	52.6%	89.6%	NA	NA	NA
	Soft tissue edema	100.0%	73.1%	NA	NA	NA
	Bone destruction	47.4%	92.5%	NA	NA	NA
	Fistula	47.4%	100%	NA	NA	NA
6. He et al. (2014) [31]	MRI findings (Thickened hyperintense synovium, extracapsular soft-tissue and bone edema, local lymphadenopathy, and extracapsular collections)	94.4%	97.4%	94.4%	97.4%	93.1%
Total knee arthroplasty						
7. Li et al. (2016) [32]*	lamellated hyperintense synovitis	78.3%	98.8%	94.7%	94.4%	94.4%
8.Plodkowskiet al (2013) [33]*	lamellated hyperintense synovitis	86%	87%	88%	86%	85.7%

PPV: positive predictive value; NPV: negative predictive value; NA: not applicable; RNS: ratio of nodal size; RNN: ratio of node number; DNS; difference of nodal size; DNN: difference of node number

*Data shown are for reader 1 or A in the original study

Synovitis is common in patients with hip or knee prostheses, and the lamellated hyperintense synovitis (LHS) is the most suggestive MRI sign of PJIs in THA [26, 28, 31] or TKA [32, 33] patients. Reasonable reliability results were found regarding LHS, with an interrater reliability of (K, 0.76–0.907) and interrater reliability of (K, 0.44–0.89) [26, 28, 32, 33]. The sensitivity and specificity for diagnosing PJIs by LHS on MRI varied between 26.3% and 86% for sensitivity and between 84% and 98.8% for specificity. The diagnostic accuracy of LHS ranged from 74.8 to 94.4% [26, 28, 32, 33].

Edema, including bone edema [26, 31], extracapsular edema [26, 31], capsule edema [27], intramuscular edema [27], and adjacent soft tissue edema [29–31] had a high correlation with the clinical diagnosis of PJIs. Interrater reliability was almost perfect for bone edema (K = 0.927) [26], extracapsular edema (K, 0.905–0.923) [26], capsule edema (K = 0.88) [27], intramuscular edema (K = 0.73–0.88) [27], and adjacent soft tissue edema (K = 0.955) [29]. The sensitivity of edema on MRI for PJI was 68.4 − 100%, and the specificity for diagnosing PJIs by edema on MRI was 73.1 − 95%. The diagnostic accuracy of edema ranged from 79.8 to 93% [26, 27, 29–31].

The MRI appearance of extracapsular collection (or fluid collection) [26, 27, 31] was suggestive of an infected arthroplasty implant. Interrater reliability was almost perfect (K, 0.905–0.923) or substantial (K = 0.68) for extracapsular collection. These articles reported the sensitivity and specificity values of 28–58% and 77.8–98%, respectively [26, 27]. The diagnostic accuracy of extracapsular collection ranged from 68.9 to 85% [26, 27].

A correlation was found between reactive lymphadenopathy (or nodal indices) on MRI and PJIs [26, 27, 29]. Results demonstrated excellent reliability for lymphadenopathy (ICC = 0.98, K, 0.844–0.99) [26, 29]. The sensitivity and specificity of the diagnoses varied between 78.9 − 93% and 47 − 87.7% [26, 29]. The diagnostic accuracy of lymphadenopathy ranged from 70 to 93.1% [26, 29].

Details of other MRI signs of PJIs are shown in Tables 2 and 3.

## Discussion

The present study aimed to systematically review the role of MRI in the assessment of infected joint prostheses for THA or TKA patients. The main findings suggest that MRI is capable of identifying suspected periprosthetic joint infection, but the definition of specific MRI features related to PJIs diagnosis lacks consensus and standardization.

All included articles were published in the last 8 years, with a rapid rise in published articles per year over time, especially in 2020–2021. The publication trend indicated that MRI assessment of PJIs is currently a research focus. MRI of metallic joint arthroplasty implants needs modified and advanced MRI pulse sequences to eliminate vast metal artifacts between the implant components and the surrounding soft tissues [23, 24]. High performance of 1.5 T MRI system is suited for achieving this function of substantial reductions in artifacts around metallic implants [23]. Hence, MRI is increasingly recognized as a noninvasive and valuable tool in the assessment of patients with problematic arthroplasty [19, 20, 43].

When inconsistent laboratory tests or nonspecific clinical symptoms are found, distinguishing between aseptic and septic implant failure remains imperfect and challenging [13, 14]. The clinical manifestation of PJIs includes the chronic, acute, low-grade, and high-grade implant infections. To date, there is no consistent diagnostic standard for PJIs in clinical practice [18, 19]. Among the aforementioned criteria, none recommend MRI as a diagnostic test for PJIs. In addition, there is another problem that conclusions of different studies on the diagnostic value of MRI for PJIs are not exactly the same. For example, Albano et al. considered conventional MRI features to have limited accuracy detecting total hip arthroplasty (THA) patients with PJIs [26], but other studies indicated the assessment of MRI findings facilitated the diagnosis of PJIs in THA or total knee arthroplasty (TKA) patients [27–33]. Possible considerations included the following: (1) very little evidence has been released on the diagnostic value of MRI for PJIs, and standardized specific MRI diagnostic features for PJIs are inconsistent and multifarious [19]. Due to the complicated anatomical structure of the hip joint, the extraction of typical MRI features on PJIs is difficult. (2) Some problems in the retrospective studies might result in serious bias risks. Most relevant articles that have been published are retrospective [26–30, 32, 33]. Because the retrospective nature of the study, it might lead to high selection bias and the possibility that the diagnostic value was falsely calculated. For example, the control group in some studies did not manifest characteristics of PJIs, but a possible low-virulence infection could not be excluded in a timely manner. (3) A periprosthetic mechanical stress reaction in MRI cannot be distinguished well from PJIs; in other words, a single positive MRI feature cannot be exclusive for implant infections [44]. (4) MRI is not extensively utilized to diagnose PJIs in clinical practice because of limitations such as high cost, long acquisition time, complex image postprocessing, and operator dependence.

Although MRI itself has the above inevitable limitations, the intrinsic multiparametric nature of MRI is conducive to achieving qualitative grading of bone destruction, synovitis, soft tissue edema, fluid collection, periosteal reaction, or lymphadenopathy, without ionizing radiation [25]. In this study, some MRI features, such as lamellated hyperintense synovitis, edema, fluid collection, or lymphadenopathy, were valuable diagnostic imaging findings. Diagnostic properties were found in terms of sensitivity, specificity, PPV and NPV (26.3 − 100%, 47 − 98%, 46 − 94.7% and 73.8 − 98%) with satisfactory accuracy (63.9 − 94.4%) and adequate reliability. Standardization is challenging, but a unique metric for the evaluation of PJIs as well as a standardized MRI protocol should be strenuously achieved, allowing MRI criteria of PJIs to be used in some suspected infections of patients who are difficult to diagnose.

Some inherent limitations included the following: (1) Collecting large-scale populations with PJIs in clinical practice is difficult, and only 206 patients with PJIs were included in this study. (2) The included studies showed statistical homogeneity and a high risk of bias, so it is improbable to perform a meta-analysis and categorize standardized MRI features for PJIs; (3) Due to the design limitations of the included studies, the diagnostic value of MRI for different types of PJIs was not clear. (4) Most included articles were retrospective designs which might result in serious variation and bias risk. Some larger prospective studies should be conducted to evaluate standardized MRI features for PJIs diagnosis in the future.

In conclusion, there is preliminary evidence that MRI has a noteworthy value of distinguishing suspected PJIs in patients with TKA or THA, but the definition of specific MRI features related to PJIs diagnosis lacks consensus and standardization. Large-scale studies with robust quality were required to help make better clinical decisions in the future.

### Electronic supplementary material

Below is the link to the electronic supplementary material.

Supplementary Material 1

## Data Availability

The datasets used and analyzed during the current study are available from the corresponding author on reasonable request.
